# Up-regulation of hexokinase II contributes to rituximab-chemotherapy resistance and is a clinically relevant target for therapeutic development

**DOI:** 10.18632/oncotarget.23425

**Published:** 2017-12-19

**Authors:** Juan J. Gu, Anil Singh, Kai Xue, Cory Mavis, Matthew Barth, Vivek Yanamadala, Peter Lenz, Michael Grau, Georg Lenz, Myron S. Czuczman, Francisco J. Hernandez-Ilizaliturri

**Affiliations:** ^1^ Department of Medicine, Roswell Park Cancer Institute, Buffalo, New York, USA; ^2^ Department of Immunology, Roswell Park Cancer Institute, Buffalo, New York, USA; ^3^ Department of Pediatric Oncology, Roswell Park Cancer Institute, Buffalo, New York, USA; ^4^ Department of Medical Oncology, Fudan University Shanghai Cancer Center, Shanghai, China; ^5^ Department of Physics, Philipps-University, Marburg, Germany; ^6^ Department of Medicine A, Hematology, Oncology and Pneumology, University Hospital Münster, Münster, Germany; ^7^ Cluster of Excellence EXC 1003, Cells in Motion, Münster, Germany; ^8^ Celgene, Summit, New Jersey, USA

**Keywords:** hexokinase II, rituximab-chemotherapy resistance, lymphoma, glucose metabolism

## Abstract

In order to identify cellular pathways associated with therapy-resistant aggressive lymphoma, we generated rituximab-resistant cell lines (RRCL) and found that the acquirement of rituximab resistance was associated with a deregulation in glucose metabolism and an increase in the apoptotic threshold leading to chemotherapy resistance. Hexokinase II (HKII), the predominant isoform overexpressed in cancer cells, has dual functions of promoting glycolysis as well as inhibiting mitochondrial-mediated apoptosis. We found that RRCL demonstrated higher HKII levels. Targeting HKII resulted in decreased mitochondrial membrane potential, ATP production, cell viability; and re-sensitization to chemotherapy agents. Analyzed gene expression profiling data from diffuse large B-cell lymphoma patients, high-HKII levels were associated with a shorter progression free survival (PFS) and/or overall survival (OS). Our data suggest that over-expression of HKII is associated with resistance to rituximab and chemotherapy agents in aggressive lymphoma and identifies this enzyme isoform as a potential therapeutic target.

## INTRODUCTION

The need to develop novel therapeutic strategies to treat relapsed/refractory aggressive lymphoma was delineated by the results of the prospective multicenter phase III Collaborative trial in relapsed aggressive lymphoma (CORAL) study [1–3]. Scientific efforts need to be focused in defining the resistance pathways utilized by lymphoma and identifying novel therapeutic strategies. To this end we developed several rituximab-resistant cell-line (RRCL) models and found that the acquirement of rituximab resistance also leads to resistance to multiple chemotherapy agents commonly used to treat B-cell lymphoma [4, 5]. Perhaps related to the acquirement of rituximab-chemotherapy resistance observed in our cell lines, we found a de-regulation of pro-apoptotic (e.g. loss of Bak/Bax), anti-apoptotic (e.g. up-regulation of Mcl-1, Bcl-XL), and inhibitor of apoptosis proteins [IAP] (e.g. up-regulation of survivin, livin) [5–7]. RRCL were found to have a high mitochondrial potential (ΔΨm), partially explained by the de-regulation of Bcl-2 family members [5]. Oligomerization of Bax and Bak is necessary for activation of the mitochondrial outer membrane permeability (MOMP) and subsequent release of cytochrome C following cytotoxic stress [8]. MOMP activation marks the commitment of cells to die by either caspase-dependent or -independent mechanism(s) and is tightly regulated by other partners such as the permeability transition pore complex (VDAC) or p53 [9, 10].

Cancer cells alter their MOMP to resist the cytotoxic effects from the host immune surveillance cells and/or the toxic effects of therapeutic interventions. As a consequence, the cellular metabolism shifts from aerobic to anaerobic glycolysis in order to generate adenosine triphosphate (ATP) (Warburg effect) [11]. In an attempt to maintain adequate ATP levels and meet their energy requirement, those cancer cells require to maintain a higher glucose uptake rate [12, 13]. Up-regulation of hexokinase (HK) is essential to maintain the Warburg effect in cancer cells. HK is the first step rate-limiting enzyme of the glycolytic pathway and mediates the phosphorylation of glucose to glucose-6-phosphate (G-6-P). Among four HK isoforms (HKI-IV), HKII has a 100-fold higher affinity for glucose than the others and is localized either free in the cytosol or bound to the mitochondrial outer membrane [14, 15]. HKII, the predominant isoform overexpressed in cancer cells, has a catalytic (C-terminal domain) and a binding domain (N-terminal domain) [16]. The catalytic domain of HKII promotes glycolysis while the binding domain interacts with VADC, protects the tumor cell from MOMP, and inhibits mitochondrial-mediated apoptosis [17, 18]. Patra *et al.* demonstrated that HKII was required in the development and maintenance of a K-ras- or ErbB-2 -driven lung cancer and breast cancer, respectively [19]. While germ line deletion of HKII causes early embryonic lethality, Patra *et al.* also demonstrated that HKII deletion in adult mice was well tolerated and the phenotype of HKII deficient mice was similar to controls [19]. Together these data leads us to postulate that: HKII/VDAC interactions may play a role in resistance to rituximab-chemotherapy and that targeting HKII is an attractive therapeutic intervention in DLBCL.

Here, we compared the intact mitochondrial membrane potential (MMP), MOMP following mitochondrial disruption, ATP production (total, cytoplasm and mitochondrial counterparts), glycolytic metabolism of RRCL with their parental cell lines and investigated the role of overexpression of HKII in drug resistance. We found that RRCL that developed concomitant resistance to multiple chemotherapy agents (referred in this manuscript as therapy resistant cell lines [TRCL]) showed higher intact MMP, repressed MOMP, enhanced ATP production and glycolysis mediated by HKII. Inhibition or gene silencing of HKII in the preclinical setting enhanced MOMP, reduced ATP production, and partially re-sensitized TRCL to chemotherapy. Using metformin, a weak physiologic HKII inhibitor, reduced HKII expression, decreased HKII/VDAC association. We also analyzed patient data and found that HKII expression is a prognostic biomarker to predict progression-free survival (PFS) and overall survival (OS) in DLCBL patients. This is the first in the literature report that expression of HKII contributes to drug resistance in the preclinical setting, and that it may have utility as a biomarker to predict survival in DLBCL in the clinical setting. HKII specific inhibition might represent a novel therapeutic approach in aggressive B-cell lymphoma.

## RESULTS

### Acquirement of resistance to rituximab and chemotherapy agents is associated with an elevated MMP and an increase in glycolysis

**Figure 1:**
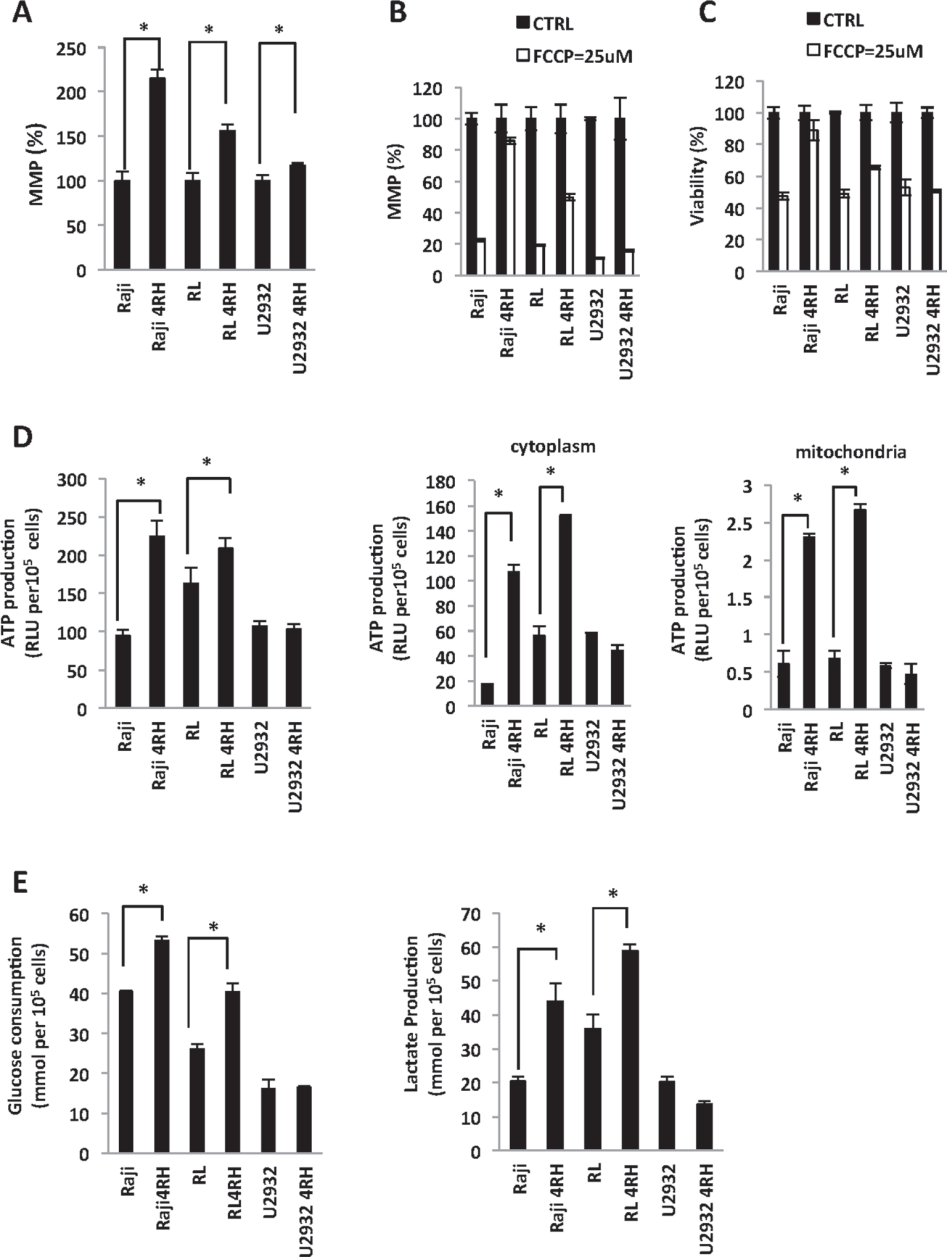
Differences in the mitochondria membrane potential (MMP) and glucose metabolism between rituximab-chemotherapy sensitive and resistant cell lines (**A**) Therapy resistant (resistant to rituximab and chemotherapy drugs) cell lines (TRCL = Raji 4RH; RL 4RH) exhibited a higher MMP than rituximab sensitive (RSCL or rituximab-resistant (RRCL = U2932 4RH) cell lines). Briefly, 5 × 10^5^ cells were pre-stained with tetraethylbenzimidazolylcarbocyanine iodide (JC-1) (1 μM) for 1 h, washed once with media and cultured for another 24 hrs. MMP was detected by the red (544/590 nm)/green (488/538 nm) fluorescence intensity ratio using a Fluoroskan. Data for each resistant cell line was normalized to their respective RSCL. (**B**) Carbonyl cyanide-*4*-(trifluoromethoxy) phenylhydrazone (FCCP) more greatly reduced the MMP in RSCL, RRCL but not in TRCL. Lymphoma cells were pre-incubate with JC-1 in 37°C for 1 hour. After wash away the JC-1, cells were exposed to FCCP (25 μM) for 48 h and MMP was measured. The data for each cell line was normalized to each untreated control cell line. (**C**) RSCL and RRCL are more sensitive to FCCP than RTCL. Cell viability was detected by Presto Blue assay after exposed to FCCP (25 μM) for 48 hrs. (**D**) Differences in ATP generation between TRCL (i.e. increased) and RSCL or RRCL. Total, cytoplasmic and/or mitochondrial ATP was determined using the Cell Titer Glo assay. Cellular and mitochondria compartment were isolated using the Mitochondrial Isolation kit. (**E**) TRCL (Raji 4RH and RL 4RH) exhibit a higher glucose consumption and lactate acid production than RSCL (Raji, RL, U2932) or RRCL (U2932 4RH). Briefly, 5 × 10^5^ cells were cultured in media for 48 hrs. Glucose consumption and lactic acid production were measured using the glucose and lactate assay kits. Experiments were performed in triplicates in three separate occasions. Each bar represents of mean ± SD. Asterisks (^*^) indicate a significant (*p* < 0.05) difference between sensitive and resistant cells at a given time point.

Previously, we demonstrated that acquirement of a resistant phenotype to rituximab and chemotherapy agents (TRCL), but not rituximab alone (RRCL), exhibited a deregulation of Bax and Bak contributing partially to their resistant phenotype to chemotherapy agents [5]. Bax, Bak, and other members of the Bcl-2 family proteins regulate the MOMP and indirectly may alter the cellular metabolism [20–23]. Therefore, we studied changes in the MMP and cellular metabolism between RSCL, RRCL, and TRCL. TRCL, but not RRCL, was associated with an increase in MMP (Figure 1A). To further characterize differences in MMP between TRCL, RRCL and RSCL, we exposed cells to FFCP (25 μM), a protonophore that uncouples the oxidative phosphorylation in the mitochondria and depolarize the mitochondrial membrane. A decrease in the MMP after exposure to FFCP was observed in RSCL (Raji, RL and U2932 cells), RRCL (U2932 4RH), and to a much lesser degree in TRCL (Raji 4RH and RL 4RH) (Figure 1B). Of interest, exposure of TRCL (Raji 4RH) to FFCP did not reduce the MMP even when higher doses of FFCP (200 μM) were used (data not show). Reduction of MMP following FFCP exposure resulted in a more decrease in cell viability in RSCL, RRCL than TRCL (Figure 1C). Together these data indicates that TRCL have a higher MMP when compared to RSCL or RRCL.

Subsequently, we explored differences in glucose metabolism and energy production (ATP) between lymphoma cells with high (TRCL) or low (RSCL and RRCL) MMP. In resting conditions, TRCL generated more ATP than RSCL or RRCL in the total, cytosol, or mitochondrial compartments (Figure 1D). Moreover, TRCL had a higher consumption of glucose and lactic acid production when compared to RSCL or RRCL (Figure 1E). Our data suggest that TRCL repressed their MOMP and altered their MMP, shifted their metabolism to anaerobic glycolysis.

### Hexokinase II (HKII) is up-regulated in TRCL

**Figure 2:**
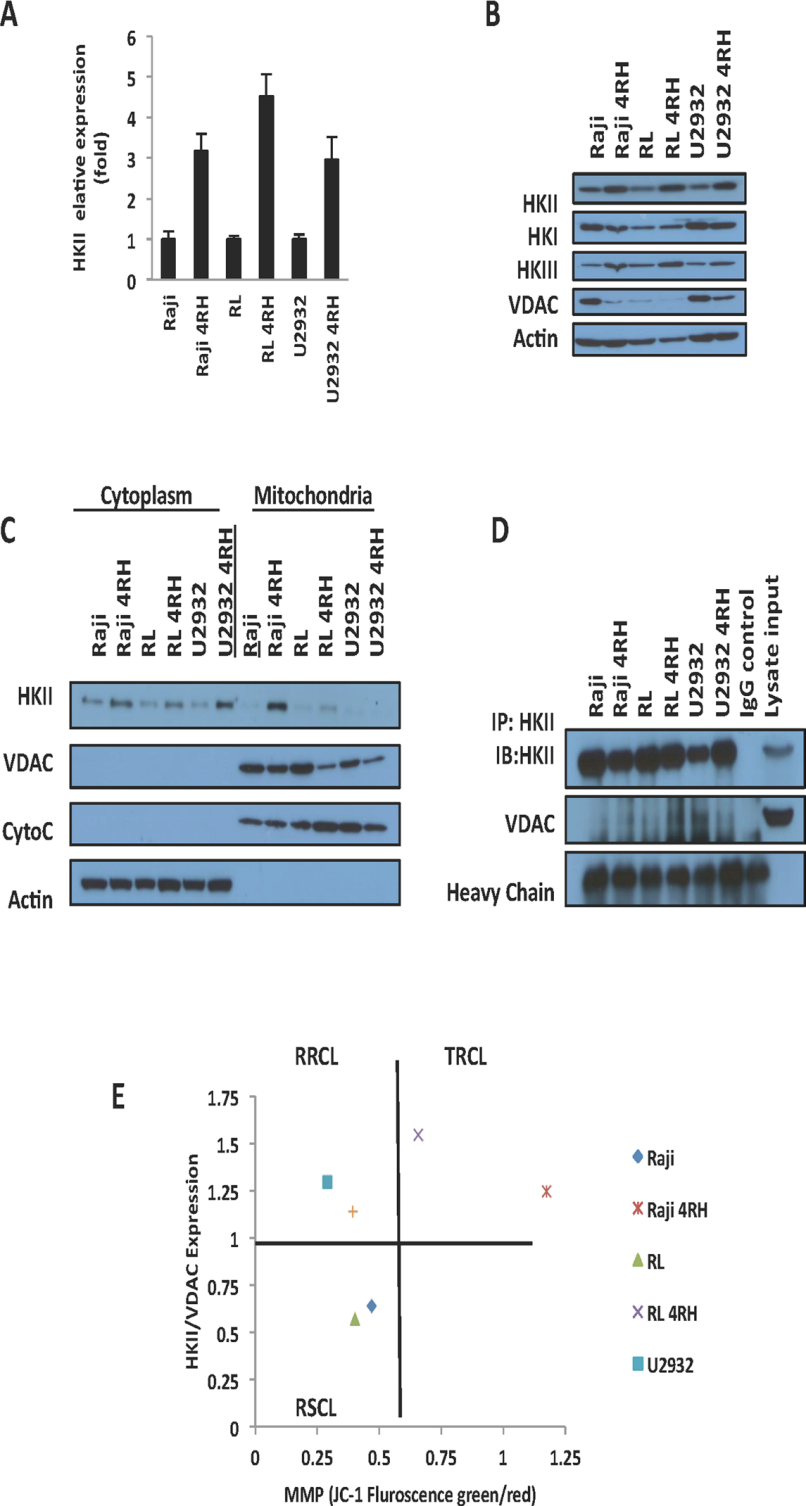
HKII is up-regulated in therapy resistant cell lines (TRCL) (**A**) Quantitative real time-polymerase chain reaction (PCR) demonstrate an increase in HKII mRNA in TRCL (Raji 4RH and RL 4RH) and to a lesser degree RRCL (U2932 4RH) when compared to rituximab sensitive cell lines (RSCL, Raji, RL, and U2932). HKII mRNA levels were normalized to actin. (**B**) Immunoblotting demonstrated an up-regulation of HKII in TRCL and to a lesser degree in RRCL. No changes in HKI or HKIII were observed. In addition, TRCL exhibited lower levels of VDAC. (**C**) Increased levels of HKII were observed in the cytoplasmic and mitochondrial cellular fragments of TRCL or RRCL when compared to RSCL. Mitochondrial or cytoplasmic fractions were isolated using the cytoplasm and mitochondrial separation kit from 20 × 10^6^ cells. Subsequently, 50µg of protein for each sample were loaded into a 12% SDS gel and were subjected to Western blotting. Localization and expression of HKII was determined using specific monoclonal antibodies. Actin was used as a cytoplasmic loading control. Cytochrome C were used as mitochondrial loading controls. (**D**) An increase in protein-protein interaction between HKII and VDAC was observed in TRCL. HKII was immunoprecipitated using a monoclonal HKII antibody. Subsequently antibody-bound complexes were submitted to immunoblotting using antibodies against HKII and VDAC. Mouse IgG and Raji cell lysates were used as input control. Heavy chain was used as loading control. Experiments were repeated in triplicates. (**E**) Correlation between HKII/VDAC expression ratio and MMP as determined by Western blotting and JC-1 staining respectively in TRCL, RRCL and RSCL. Each dot represents the average of three independent experiments.

Previous work demonstrated that several key enzymes of the glycolysis are up-regulated in drug resistant cells including hexokinase (HK), phosphofructokinase (PFK), pyruvate kinase M2 isoform (PKM2), lactate dehydrogenase A (LDHA), and pyruvate dehydrogenase kinase 1 (PDK1) [12, 24, 25]. Distinctly, HKII is known to affect the MMP and play a pivotal role in glycolysis. Using qPCR we found that TRCL had higher levels of HKII mRNA levels than RSCL or RRCL (Figure 2A). In addition, Western-blotting studies further confirmed that TRCL up-regulated HKII expression at the protein level (Figure 2B). Potentially related to the chemotherapy-resistant phenotype observed in these cells, VDAC, a known gatekeeper of the MMP that interacts with HKII, was found to be down-regulated in TRCL by Western blotting. On the other hand, no differences in the expression of other HK isoforms (HKI and HKIII) were detected between TRCL and RSCL/RRCL.

HKII can be found free in the cytosol or bound to the mitochondrial outer membrane where it represses the MMP [14]. To determine differences in HKII expression/localization between RSCL, RRCL and TRCL we isolated cytoplasm and mitochondrial subcellular fractions and determined HKII, VDAC, and Cytochrome C expression by Western blotting. In TRCL and to a lesser degree RRCL but not in RSCL, HKII was up-regulated in both cellular fractions (Figure 2C). In addition, HKII co-immunoprecipitation studies demonstrated that, HKII-VDAC protein-protein interactions were higher in TRCL than in RRCL or RSCL (Figure 2D). To determine the correlation between HKII/VDAC protein-protein interactions and MMP, we quantified HKII and VDAC expression levels using Western blotting and plotted the HKII/VDAC relative density ratio (RDR) against the MMP in each RSCL, RRCL or TRCL (Figure 2E). Using this approach, our cell lines were categorized in three groups: Cells with a low HKII/VDAC RDR and low MMP (Included all the RSCL), cells with an elevated HKII/VDAC RDR and low MMP (RRCL) and cells with an elevated HKII/VDAC RDR and high MMP (All the TRCL) (Figure 2E).

### TRCL are more sensitive to HKII inhibition than RSCL

**Figure 3:**
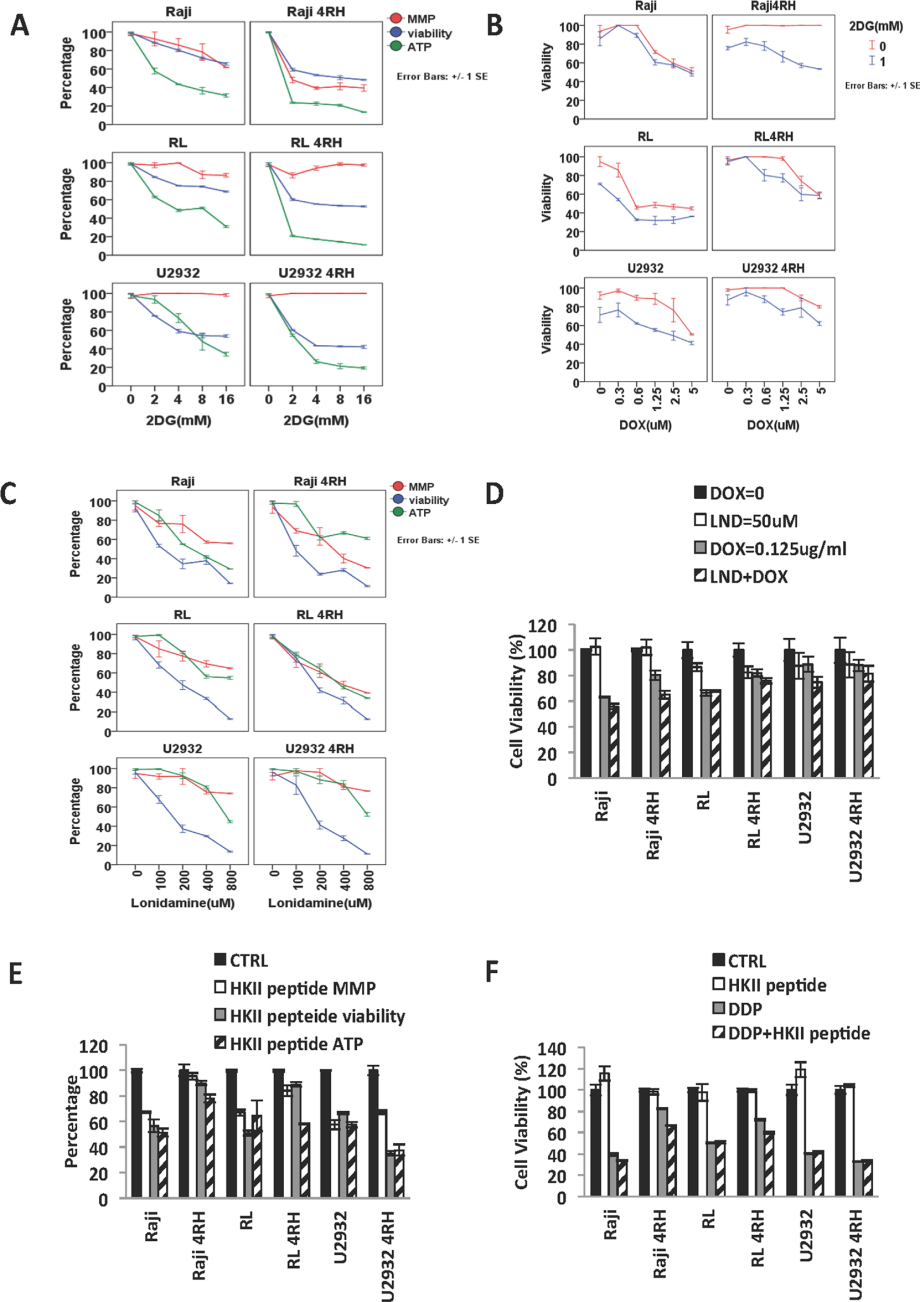
Distinct effects of hexokinase inhibition in therapy-resistant (TRCL), rituximab-resistant (RRCL) and rituximab-sensitive cell lines (RSCL) (**A**) Inhibition of glucose uptake by 2DG decreased the cell viability and ATP reduction in TRCL, RRCL and to a lesser degree RSCL. Cells were incubated in escalating dosage of 2DG for 48 h. Changes in cell viability, ATP reduction and MMP were detected by Presto blue, cell titer Glo, and MMP assays respectively. (**B**) Inhibition of glucose uptake enhanced the cytotoxic effects of doxorubicin in TRCL, RRCL, and RSCL. Initially, 5 × 10^5^ cells were exposed to 2DG (1 mM) or control for 24 hrs. Subsequently control or 2DG exposed cells were exposed to escalating doses of doxorubicin for 48 hrs. Changes in viability were determined by Presto blue. (**C**) Lonidamine (LND), a HKII inhibitor, depleted cellular ATP levels, lowered MMP and decreased cellular viability in a panel of lymphoma cell lines. (**D**) Inhibition of HKII by LND enhanced the killing effect of doxorubicin in in TRCL, RRCL, and RSCL compared to chemotherapy alone. (**E**) Inhibition of HKII-VADC binding domain using a HKII binding peptide resulted in variable changes in ATP reduction, MMP, and cell viability of lymphoma cells. B-cell lymphoma cell lines were exposed to the HKII binding peptide (50 µM) for 48 hrs. Changes in mitochondrial potential (MMP), cell viability, and ATP generation were determined by MMP, Presto blue, and cell titer glow assays respectively. (**F**) Disruption of HKII-VADC binding using the HKII VDAC binding domain peptide was associated with partial re sensitization of therapy resistant cell lines (TRCL, Raji 4RH and RL 4RH) to cisplatin. Lymphoma cells were exposed to the HKII-VADC binding peptide (50 µM) 24 hrs, and subsequently exposed to escalating doses of cisplatin. Changes in cell viability were determined using the Presto blue assay. Data represent the mean ± SD of three independent experiments performed in triplicates. Asterisks (^*^) indicate a significant (*p* < 0.05) difference between sensitive and resistant cells at a given time point.

Inhibition of glycolysis, in particular by targeting HKII is an attractive but challenging anti-cancer therapeutic strategy [26]. Based on the changes in glucose metabolism and HKII/VDAC levels observed in our TRCL, we further studied the effects of disrupting the glucose uptake/metabolism in our panel of cell lines. We exposed RSCL, RRCL and TRCL to 2DG, a competitive inhibitor of glucose. 2DG is converted into 2-DG-P by HKII but it cannot be further catabolized by the glycolytic pathway and accumulates in the cytoplasm, depletes ATP reserves, and eventually inhibits HKII [27–29]. *In vitro* exposure of TRCL, but not RSCL or RRCL, to 2DG resulted in a decrease in glucose uptake. In addition, *in vitro* exposure to 2DG decreased the ATP content and the viability, but did not affect the MMP of TRCL (and to a lesser degree RRCL and RSCL) (Figure 3A). Inhibition of glucose uptake by 2DG enhanced the cytotoxic effects of doxorubicin in TRCL, RRCL, and RSCL (Figure 3B).

To further study the role of HKII in the viability of RSCL, RRCL and TRCL, we evaluated the anti-tumor activity of lonidamine (LND), a hexokinase inhibitor. *In vitro* exposure of B-cell lymphoma cells to escalating doses of LND (0–800 µM) resulted in a dose-dependent decrease in MMP, ATP content and cell viability (Figure 3C). Inhibition of HKII by LND enhanced the killing effects of doxorubicin in TRCL, RRCL, and RSCL (Figure 3D). Similar findings were observed when HKII-VDAC protein-protein interaction was disrupted in RSCL, RRCL, and TRCL exposed to an HKII-VDAC peptide. The synthetic peptide was designed to disrupt the interaction of N-terminal of HKII and VDAC. *In vitro* exposure of RSCL, RRCL, and TRCL to a HKII-VDAC competitive peptide (50 µM) decreased ATP content, MMP and cell viability (Figure 3E). In addition, disruption of HKII-VDAC interaction reversed the acquired resistance to chemotherapy agents (doxorubicin) in TRCL (Figure 3F). All these indicated that blockage of HKII by different approaches enhanced MOMP, reduced ATP production, and partially re-sensitized TRCL to the chemo-reagent.

### Metformin, Idelsilib and temsirolimus decreased HKII levels; lower the mitochondrial potential and enhanced chemotherapeutic drugs of diffuse large B-cell lymphoma

**Figure 4:**
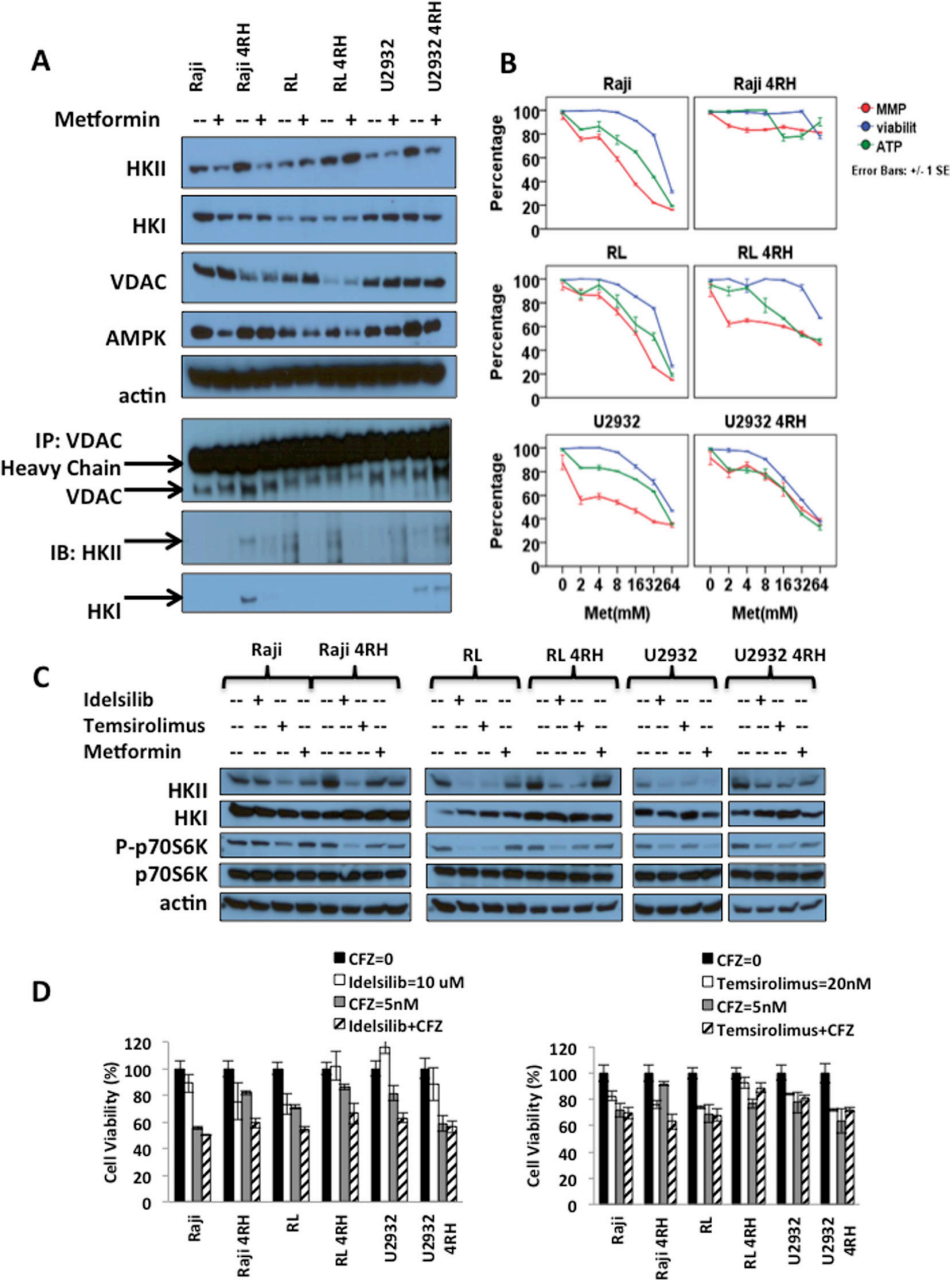
Effects of metformin (an HKII inhibitor) and other PI3K-Akt-mTOR inhibitors in B-cell lymphoma (**A**) *In vitro* exposure of therapy-resistant (TRCL), rituximab-resistant (RRCL), and rituximab-sensitive cell line (RSCL) to metformin led to a decrease in HKII levels. Lymphoma cell lines were exposed to metformin (8 mM) for 24 hrs. Changes in HKII, HKI, VADC and AMPK were determined by Western blotting. Actin was used as loading control. In addition, HKII-VDAC protein-protein interaction after metformin treatment was showed by VDAC immunoprecipitation assay described previously. (**B**) *In vitro* exposure of TRCL, RRCL and RSCL to metformin resulted in anti-tumor activity. Briefly, 5 × 10^5^ cells were exposed to escalating doses of metformin for 24 hrs. Changes in mitochondrial potential (MMP), cell viability, and ATP generation were determined by MMP, Presto blue, and cell titer glow assays respectively. Each data represents of mean ± SD of three independent of experiments. (**C**) *In vitro* exposure of therapy-resistant (TRCL), rituximab-resistant (RRCL), and rituximab-sensitive cell line (RSCL) to PI3K inhibitor idelsilib, mTOR inhibitor temsirolimus or metformin led to a decrease in HKII levels. Lymphoma cell lines were exposed to idelsilib (10 uM), temsirolimus (20 nM) or metformin (8 mM) for 24 hrs. Changes in HKII, HKI, phosph-p70S6K and p70S6K were determined by Western blotting. (**D**) Inhibition of either PI3K or mTOR enhanced the killing effect of carfilzomib in TRCL, RRCL, and RSCL compared to chemotherapy alone at 72 hours.

Metformin is an agonist of the adenosine monophosphate-activated protein kinase (AMPK) that plays a pivotal role in cellular metabolism and B-cell development. It has been demonstrated that AMPK inhibits the mammalian target of rapamycin (mTOR) and lowers HKII levels in cancer pre-clinical models [30]. Clinically, metformin improves the outcome of diabetic patients with solid tumor malignancies, either alone or combined with chemotherapy, indicating its potential role in cancer therapy [31]. *In vitro* exposure of RSCL, RRCL and TRCL lymphoma cells to metformin decreased AMPK and HKII levels. No changes in HKI or VDAC levels were observed following metformin exposure (Figure 4A). In addition, metformin exposure inhibited HKII-VDAC protein-protein interaction (Figure 4A). *In vitro* exposure of RSCL, RRCL and to a lesser degree TRCL resulted in a decrease in the MMP, ATP content, and cell viability (Figure 4B). Moreover, we also found PI3K inhibitor idelsilib or mTOR inhibitor temsirolimus decreased HKII levels in RSCL, RRCL and TRCL. But HKI level were observed no change after drug exposure (Figure 4C). Idelsilib or termsirolimus enhanced the killing effects of proteasome inhibitor carfilzomib in TRCL, RRCL, and RSCL (Figure 4D).

### HKII role in the cell viability of lymphoma cells and contributes to chemotherapy resistant in B-cell lymphoma

**Figure 5:**
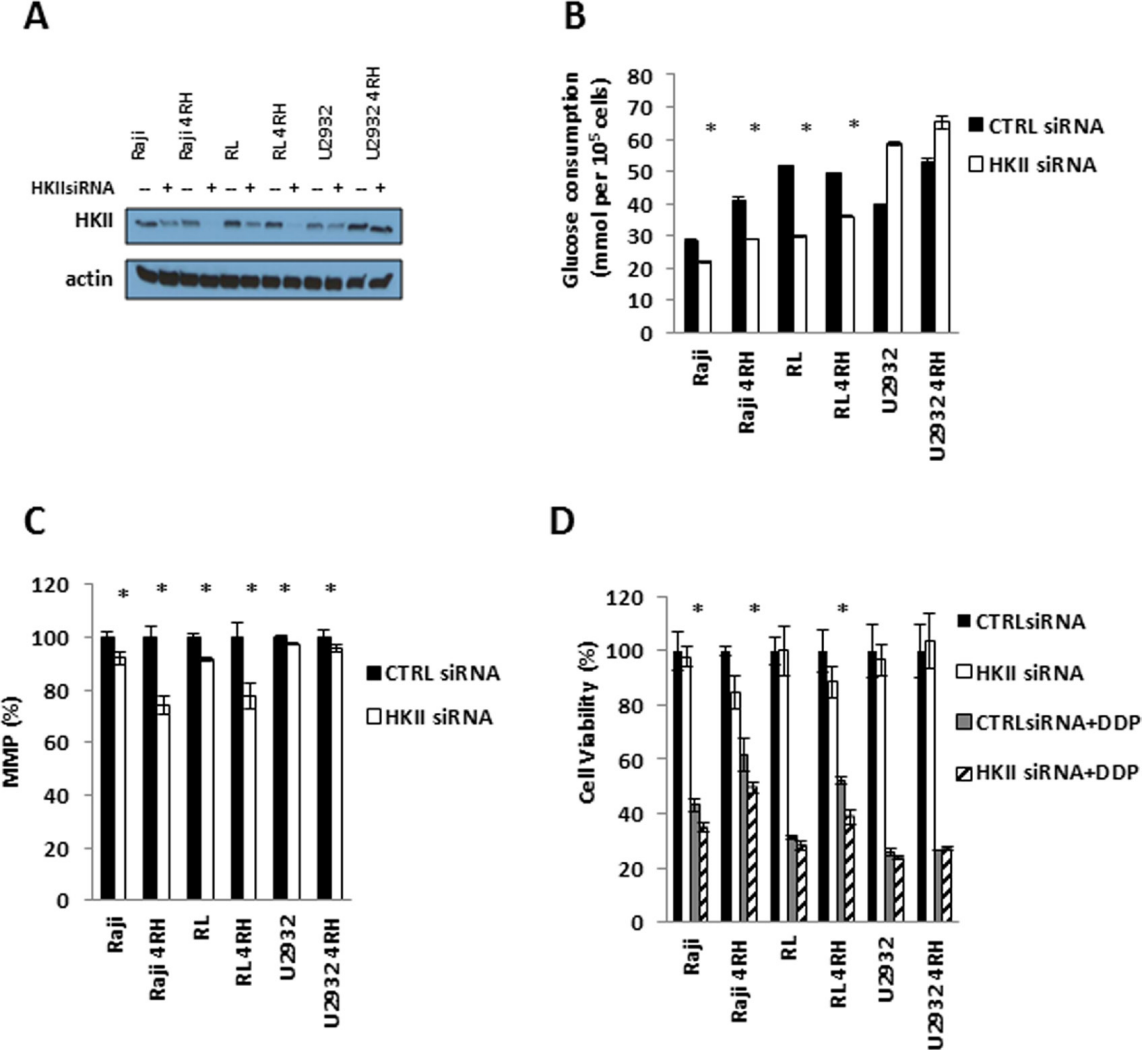
HKII is necessary in TRCL (**A**) Endogenous HKII was knocked down using OnTARGET plus SMART pool siRNA against HKII. Efficient knock down was confirmed by western blotting. (**B**) Following HKII siRNA knock down, changes in glucose consumption were measured using the glucose consumption assay. (**C**) Gene silencing of HKII resulted in a reduction in mitochondrial membrane potential (MMP). (**D**) Subsequently, HKII siRNA or control siRNA transfected cells were exposed to different doses of cisplatin for 48 hrs. Changes in cell viability were determined using the Presto blue assay. Asterisk (^*^) represents *P* < 0.05. Each bar represents of mean ± SD of three independent of experiments.

To address the role of HKII in chemotherapy resistant B-cell lymphoma, control scramble small interfering RNA (CTRL siRNA) and HKII siRNA were transfected into RSCL, RRCL and TRCL to determine whether HKII has a role in drug resistance. HKII expression level was significant decreased 48 hours after transfection (Figure 5A). Glucose consumption could be inhibited and MMP decreased by transient siRNA knockdown of HKII in RSCL, RRCL and TRCL (Figure 5B and 5C). Furthermore, HKII silencing reversed the acquired resistance to chemotherapy agents (cisplatin) in TRCL (Figure 5D).

### The clinical significance of HKII expression in DLBCL

**Figure 6:**
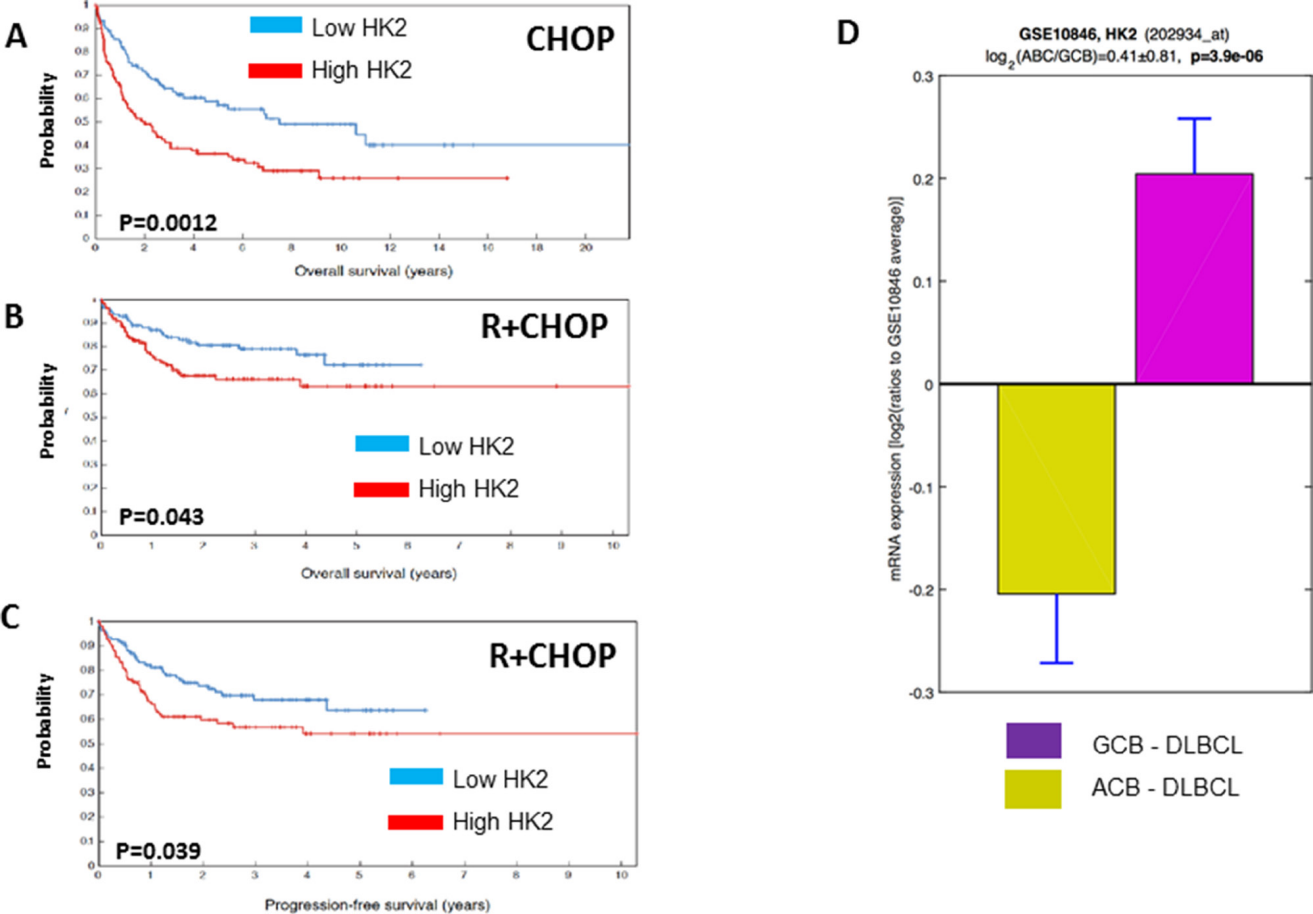
Higher levels of HKII are associated with a shorter progression free survival (PFS) or overall survival (OS) in diffuse large B-cell lymphoma Gene expression profiling (GEP) studies conducted in diffuse large B-cell lymphoma (DLBCL) patients treated with cyclophosphamide, doxorubicin, vincristine and prednisone (CHOP) or rituximab and CHOP (R+CHOP) demonstrated that higher levels of HKII are associated with a shorter OS in patients treated with CHOP (**A**) and inferior OS and PFS in DLBCL treated with R+CHOP (**B**, **C**). HKII expression was higher in ABC DLBCL than in GCB DLBCL (**D**)

To further assess the potential contribution of HKII to rituximab/chemotherapy resistance in a more clinically relevant setting, we analyzed gene expression profiling data from 401 patients with DLBCL treated with CHOP- (*n* = 181) or R-CHOP-like treatment (*n* = 220) as front-line therapy. High levels of HKII correlated with a poor prognosis in DLBCL patients. In patients receiving CHOP chemotherapy, high levels of HKII correlated with a shorter OS (*P* = 0.0012). Similarly, high-HKII mRNA levels were associated with a shorter PFS (*P* = 0.039) and OS (*P* = 0.043) in R-CHOP treated DLBCL (Figure 6). To analyze the association with DLBCL molecular subtype, we compared KHII expression between GCB-DLBCL and ABC DLBCL in the two cohorts of DLBCL patients (*N* = 401). Average HKII expression was significantly higher in the ABC DLBCL cohort (*P* = 0.000039) (Figure 6). We conducted a multivariate Cox proportional hazard model to determine the predictive value of KHII in DLBCL. Of note, is important to disclaim that IPI score was not available for DLBCL treated with CHOP. Unfortunately, KHII did not have additional predictive value on top of the molecular subtypes of DLBCL (*P* = 0.34). With respect to IPI score, there was an additional predictive value from KHII (*P* = 0.03). However this was only observed in the R-CHOP treated cohort of patients.

## DISCUSSION

The early use of rituximab as a single agent or in combination with various systemic chemotherapy regimens (i.e. CHOP) have resulted in improved response rates, duration of remission and improved overall survival in patients with DLBCL [32–40]. Thus, it is mandatory that scientific efforts have to focus on defining biological differences between responding and resistant patients as well as in identifying cellular/metabolic pathways utilized by lymphoma cells to evade immunochemotherapy. Our data suggests a pivotal role of HKII in rituximab-chemotherapy resistance and its expression is associated with an inferior outcome in DLBCL patients treated with either CHOP or R+CHOP in the front-line setting. HKII expression differs between molecular subtypes of DLBCL. We found, that KHII expression is higher in ABC DLBCL than in GCB-DLBCL. Our multivariate analysis failed to demonstrate an additional predictive value from HKII expression on top to DLBCL molecular profiling. However, HKII appears to be a suitable target for therapeutic interventions, specially in ABC DLBCL.

Fundamental differences in the cellular metabolism had been observed between normal and cancer cells by various studies. It has been well documented that cancer cells increase aerobic/anaerobic glycolysis as they exhibit a great dependency on the glycolytic pathway for ATP generation [11]. In contrast, very little is known how disease progression following immunochemotherapy alters the metabolism of cancer cells. In our rituximab-resistance model, we detected that the glucose metabolism is further altered only in TRCL but not in RRCL “only” models. TRCL, that are resistant to both rituximab and chemotherapy drugs, were found to have higher MMP and repressed MOMP. We previously reported that TRCL are associated with deregulation of pro-apoptotic and anti-apoptotic proteins that lead to concomitant chemotherapy resistance [5]. Restoration of abnormal levels of Bcl-2 family members partially reversed the resistant phenotype, suggesting the existence of alternative regulatory proteins governing the MMP and chemotherapy resistance observed in TRCL.

HKII phosphorylates glucose into glucose-6-phosphate (G-6-P) which is the first limiting reaction in glycolysis. In addition, HKII is localized in the mitochondrial membrane where it interacts with VDAC and members of the Bcl-2 family members affecting the MMP and thereby responses to apoptotic stimuli [41, 42]. Disruption of the interaction between HKII and VDAC enhance the induction of apoptosis in cancer models [43]. Over-expression of HKII has been associated with chemotherapy-resistance and poor clinical outcomes in liver, pancreatic carcinomas, ovarian and hepatocellular carcinoma [44–48]. HKII was up-regulated in our TRCL and pharmacological inhibition or gene silencing of HKII resulted in cell death and a re-sensitization to chemotherapy agents. Moreover, we discovered that over-expression of HKII was associated with a shorter PFS in DLBCL treated with R+CHOP and an inferior OS in DLBCL patients treated with CHOP- or R-CHOP-like therapy. HKII expression was higher in ABC DLBCL than in GCB DLBCL. Thus, our work identifies a potentially novel biomarker of poor clinical outcome in patients with DLBCL in the R-CHOP era and equally important a potentially novel target for therapeutic intervention. To our best knowledge this is the first report that associates rituximab-chemotherapy resistance to changes in the glycolytic pathway and HKII expression.

Metformin has been found to be an inhibitor of HKII in lung and breast cancer cell lines, primarily via inhibition of mammalian target of rapamycin (mTOR) leading to a decrease in HKII mRNA transcription [49, 50]. Epidemiological studies have demonstrated that the use of metformin during chemotherapy in type 2 diabetes patients is associated with an improved survival in breast, prostate, colorectal, and lung cancer [31]. Prior to this manuscript, no work has evaluated the effect of the concurrent use of metformin in type 2 diabetic DLBCL patients undergoing front-line immunochemotherapy. *In vitro* exposure of RSCL, RRCL and TRCL to metformin resulted in a down-regulation of HKII levels, decrease in cell viability and improvement on the cytotoxic effects of chemotherapy agents. This information suggests that pharmacological inhibition of HKII using metformin may be an attractive and inexpensive strategy to overcome intrinsic or acquired resistance to chemotherapy agents and may improve clinical outcomes. On the other hand, metformin is known to induce lactic acidosis when is use at higher doses and/or in patients with comorbid conditions (i.e. patients with poor renal function or symptomatic congestive heart failure) [51]. It is unclear if the pharmacological levels of metformin necessary for inhibition of HKII in patients can be achieved without a significant increase in lactic acidosis. We also demonstrated that pharmacological inhibition of the PI3K-Akt-mTOR pathway with idelsilib or temsirolimus decreases HKII levels and can potentiated the effects of proteasome inhibitors. Our findings provide evidence of alternative routes to decrease HKII levels with readily available targeted agents.

Alternative strategies to inhibit HKII such as: 1) down-regulation of HKII using novel and more potent mTOR inhibitors, or 2) selective inhibition of the HKII binding domain or disrupting its interaction with Bcl-2 family members or VDAC may result in significant anti-tumor activity in B-cell lymphoma. Selective targeting the catalytic and/or binding domain of HKII is a potentially viable yet challenging strategy in the future development of novel strategies to overcome resistance in the field of cancer therapeutics.

## MATERIALS AND METHODS

### Cell lines and chemicals

A panel of rituximab-chemotherapy sensitive [RSCL], and RRCL, (which were found to be chemotherapy-resistant) may also be referred to as TRCL in this manuscript. The parental, RSCL Raji (Burkitt’s lymphoma), and RL (germinal center B-cell [GCB] DLBCL) were purchased from American Type Culture Collection (ATCC, Manassas, VA). The parental U2932 (activated B-cell [ABC] DLBCL) cell line was acquired from German Collection of Microorganisms and Cell Cultures (Braunschweig, Germany). The RRCL/TRCL (Raji-4RH, RL-4RH, U2932-4RH) were created and characterized from RSCL as previously described [4, 5]. Of note, the U2932-4RH cell-line developed resistance to rituximab, but remained sensitive to the anti-tumor activity of several chemotherapy agents (RRCL). Carbonyl cyanide-*4*-(trifluoromethoxy) phenylhydrazone (FCCP) was obtained from Santa Cruz Biotechnology (Dallas, TX). 2-Deoxy-D-glucose (2DG) and metformin were purchased from Sigma-Aldrich (St. Louis, MO). The hexokinase II/voltage-dependent anion channel (HKII/VDAC) binding domain peptide was obtained from Millipore (Darmstadt, Germany) and Lonidamine was purchased from Sigma-Aldrich. Bortezomib, carfilzomib, doxorubicin, cisplatin, Idelsilib and termsirolimus were purchased from Selleckchem (Houston, TX).

### Glucose consumption, lactate acid production, and adenosine triphosphate (ATP) generation

Lymphoma cell lines were seeded (5 × 10^5^/ml cells) in phenol-free culture media and incubated for 24 hrs. The culture media was collected to measure glucose consumption and lactate production. Glucose and lactate levels were determined using a Glucose or Lactate assay kit (Biovision, San Francisco, CA) according to manufacturer instructions. Glucose consumption was determined by correcting the difference between glucose concentration in cell-free media and the supernatant collected from each cell line culture. Cell numbers were counted with an Automated Cell Counter (Invitrogen Inc, Grand Island, NY) to normalize the glucose and lactic acid concentration according to manufacturer instructions. In addition, differences in ATP production over time between RSCL, RRCL and TRCL were determined using the CellTiter Glo cell viability assay kit from Promega (Madison, WI) following the manufacturer’s instructions. Results were standardized to the number of cell/well and repeated in triplicate.

### Detection of mitochondrial potential changes

We assessed differences in the baseline mitochondrial potential between RSCL, RRCL and TRCL using the tetraethylbenzimidazolylcarbocyanine iodide (JC1) dye Mitochondrial Membrane Potential Assay Kit (Abcam, Cambridge, MA). Briefly, 2.5 × 10^5^ cells were stained with JC-1 (1 µM) for 30 minutes at 37°C, excess of dye was washed off once. Subsequently, cells were seeded in 384-well plates, overlaid with control or FCCP (25 µM) and incubated for 48 h. Signal was read at 544/590 nm for high mitochondrial potential and at 488/538 nm for low mitochondrial potential using the Fluoroskan ascent LF (Thermo Fisher Scientific, Barrington, IL). The MMP was calculated using the following formula: MMP = high mitochondrial potential/low mitochondrial potential and then normalized to each parental cell or control cell (when exposed to FCCP).

### Western blot of key regulators of the MMP

In order to study the significance of HKII and VDAC in rituximab-chemotherapy resistance we studied differences in the expression of HKI, HKII, HKIII and VADC by Western blotting. Primary mouse or rabbit anti-human monoclonal antibodies (mAbs) against HKI, HKII, VDAC, cytochrome C, and β-actin (loading control) were obtained from Cell Signaling Technology (Boston, MA). After adding the appropriate AP- or HRP-conjugated secondary antibody, detection was performed by Western-lightning Plus ECL chemiluminescent substrate (PerkinElmer, Waltham, MA). Mitochondrial and cytosolic fractions were separated by the mitochondrial isolation kit (Thermo Scientific, Rockford IL) according to the manufactory’s instructions. After separation, mitochondrial and cytoplasmic fractions were lysed with a 2% CHAPS and protein quantification was performed.

### Real-time quantitative PCR

HKII gene expression level in the parental and resistant cells was analyzed by quantitative real-time polymerase chain reaction (qPCR). Total RNA was extracted and converted in complementary Deoxyribonucleic acid (DNA) using the Taqman Gene Expression cells-to-CT kit (Life Technologies, Grand Island, NY). Real-time PCR reaction was carried out using PCR Master Mix (Life Technologies) on ABI-7900 platform (Applied Bio systems). HKII was amplified from cDNA using primers designed to amplify its coding region (Applied Bio systems, Hs00606086).

### Effects of targeting the PI3K-AKT-mTOR pathway in HKII expression and cell viability

RSCL and RRCL were exposed to metformin *in vitro*. Changes in HKII expression were determined by Western blotting. Changes in mitochondrial potential (MMP), cell viability, and ATP generation were determined by MMP, Presto blue, and cell titer glow assays respectively. To further evaluated the therapeutic effect of targeting the PI3K-AKT-mTOR pathway, RSCL and RRCL were exposed to idelsilib (10 nM) or temsorilimus (20 nM) for 24 hours. Changes in HKII, KHI, and PI3K downstream targets were evaluated by Western blotting. To test if targeting the PI3K-AKT-mTOR pathway and HKII levels, could enhance the anti-tumor activity of chemotherapy agents, RSCL and RRCL were exposed to idelsilib or temsirolimus alone or in combination with doxorubicin, cisplatin, bortezomib, or carfilzomib for 72 hrs. Changes in cell viability were detected by alamar blue reduction.

### Transfection of HKII-specific siRNAs sMART pool

We silenced HKII using siRNA interference by using ON-TARGET plus SMART pool siRNA containing a mixture of 4 siRNAs designed to specifically target *HKII* (Dharmacon, Lafayette, CO). Non-targeting SMART pool siRNA (Dharmacon) was used as a negative control. Efficient knockdown of HKII was confirmed by Western blotting. Once conditions were optimized, RSCL, RRCL or TRCL were transfected with siRNA targeting HKII or control using an Amaxa Nucleofector (Lonza laboratories, Anaheim CA) and cells were incubated for an additional 48 hours. Differences in glucose consumption and cell viability were determined as previously described.

### HKII expression in DLBCL patients before therapy and follow-up

To further assess the contribution of HKII to rituximab/chemotherapy resistance in a more clinically relevant setting, we analyzed the gene expression profile (GEP) data set from 414 patients with DLBCL treated with CHOP- (*N* = 181) or R-CHOP-like treatment (*N* = 220) [52]. Follow-up data for 181/181 CHOP treated patients (overall survival) and for 220/233 R-CHOP treated patients (overall and progression-free survival) are available. All data were obtained from the NCBI GEO database (accession GSE10846) [53]. Subsequently, we compared differences in HKII expression between GCB-DLBCL and ABC-DLBCL using a two-tailed two-sample *t*-test. A fitted multivariate Cox proportional hazard models was performed to determine the predictive value of HKII in the context of other explanatory value for molecular interpretation of DLBCL and clinical parameters (i.e. International Prognostic Index [IPI] score).
